# Are You a Closet Dualist? Evidence From Brief Implicit Association Task

**DOI:** 10.1162/opmi.a.7

**Published:** 2025-07-26

**Authors:** Iris Berent, Alexzander Sansiveri

**Affiliations:** Department of Psychology, Northeastern University, Boston, MA, USA

**Keywords:** dualism, intuitive psychology, intuitive bias, intuitive physics, core knowledge, implicit association task

## Abstract

Do people tacitly contrast minds and bodies? To find out, here, we gauge Dualism using a brief implicit association task. Participants were asked to determine whether a target word belonged to a category-attribute pair. Categories were either body or mind; attributes captured either physical properties of bodies (e.g., object/solid) or their converse (e.g., stuff/airy). Results from five experiments showed that physical properties selectively facilitated responses only to body (but not mind). In Experiments 3–5, responses to mind were further facilitated by airy (relative to solid). Together, these results suggest that people tacitly view the mind as ethereal, distinct from the physical body. Remarkably, this was the case even in participants who explicitly rejected Dualism. Dualism, then, is an implicit bias that persists despite explicit attitudes to the contrary. These conclusions shed light on why educated Western adults contrast minds and bodies.

## INTRODUCTION

A large literature suggests that people are intuitive Dualists—they consider the mind as ethereal, distinct from the body (Barlev & Shtulman, 2021; Bloom, 2004; Musolino, 2015). The mind-body divide matters because it can derail reasoning, promoting bias and prejudice.

For instance, educated Western adults believe that, if a person’s depression “shows up” in the brain (e.g., as an activation spike), then this condition is more likely to be innate and immutable compared to a diagnosis obtained by a behavioral measure (e.g., faster response time; Berent & Platt, 2021a). Additionally, trained clinicians falsely assume that, if a disorder has a biogenetic origin, then patients are less likely to benefit from psychotherapy (Ahn et al., 2017). Finally, psychiatric conditions that are linked to the brain are considered lengthier (Lebowitz & Ahn, 2014; Lebowitz et al., 2013), less responsive to treatment (Kvaale, Haslam, & Gottdiener, 2013; Loughman & Haslam, 2018), and more stigmatizing by laypeople and patients alike (Ahn et al., 2017; Berent & Platt, 2021a; Kvaale, Gottdiener, & Haslam, 2013; Loughman & Haslam, 2018).

Other research has linked Dualism to reasoning fallacies about science. Laypeople consider brain explanations more satisfying than cognitive explanations, even when these brain explanations are plainly circular (Weisberg et al., 2008), and this bias is demonstrably linked to Dualism (Sandoboe & Berent, 2021). Laypeople further assume that certain psychological traits (e.g., basic emotions) are more likely to “show up” in the brain than others (non-basic emotions: Berent et al., 2020; thoughts: Berent et al., 2021), and that, if a trait “lights” the brain, then this trait is likely innate (Berent & Platt, 2021b; Berent et al., 2021).

These beliefs reflect a misconception, as science suggests that *all* psychological traits—innate or learned—engage the brain (Musolino, 2015; Pinker, 2002). Accordingly, a brain signature offers no evidence that the relevant trait is innate. Likewise, psychological conditions that are gleaned from a behavioral response are just as likely to engage the brain as those gleaned from a brain response; beliefs to the contrary reflect the fallacy that minds and bodies are distinct (Musolino, 2015; Pinker, 2002). That educated Western adults fall for this bias is surprising. In fact, these participants demonstrably separate bodies and minds even when they vehemently endorse Physicalism—the belief that bodies and minds are one and the same (Bering, 2002).

But it is not only Western individuals who segregate minds and bodies. Similar beliefs have been shown in small-scale societies (Astuti & Harris, 2008; Boyer, 2018; Chudek et al., 2018; Cohen & Barrett, 2008; Cohen et al., 2011; Lane et al., 2016; Slingerland & Chudek, 2011; Watson-Jones et al., 2017; Weisman et al., 2021), including ones that do not explicitly discuss the minds of others (Chudek et al., 2018), and they are further evident in young children (e.g., Bering & Bjorklund, 2004; Chudek et al., 2018; Emmons & Kelemen, 2015; Gottfried & Jow, 2003; Hood et al., 2012; Nichols, 2006).

Why, then, do people intuitively separate minds and bodies? One possibility is that Dualism arises solely by cultural transmission. While we do not doubt that cultural transmission is powerful, cultural transmission alone does not explain why Dualism is prevalent across cultures.

An alternative account asserts that Dualism is deeply grounded in human cognition. The cognitive bases of Dualism can further explain (a) why it is *prevalent* across cultures; (b) why it is *pernicious*—why it might implicitly take root even in educated adults who otherwise profess belief in Physicalism; and (c) how, precisely, do people intuitively conceive of bodies and minds.

The present research tests the latter two predictions (b–c). To be clear, *why* Dualism arises (a) is not a question we will address experimentally. We nonetheless believe it is useful to briefly consider the cognitive origins of Dualism, as doing so can explain both (b) *how* people conceive of bodies and minds and (c) why the mind-body divide might be pernicious, and possibly implicit. It is these latter two questions that are the center of this research.

### The Cognitive Origins of Dualism

Over twenty years ago, Paul Bloom (2004) proposed that Dualism arises from the clash between two systems of core knowledge: intuitive physics and mindreading.

Intuitive physics governs our initial understanding of objects (Spelke, 2022). It suggests that objects are bound, cohesive, solid, and they move only by immediate contact with other objects. Since human bodies (e.g., a hand) are bound and solid, much like a ball, bodies ought to engage our physical system, and lead people to project these physical intuitions to bodies: people should expect bodies to abide by the same physical principles that apply to objects.

But these physical expectations soon clash with those generated by a second cognitive system—mindreading (Leslie, 1987). Per the mindreading system, people’s actions ought to be caused by their mental states: their beliefs, goals and desires. So, when you see a person grab a cup of coffee, mindreading would spontaneously attribute this action to the agent’s desire (for coffee) and goal (grab the coffee).

From the perspective of intuitive physics, this event ought to be baffling. A hand, as noted, is an object (just like a ball), and objects aren’t supposed to move spontaneously (without physical contact with another object). Everyday behavior, then, ought to elicit a tacit cognitive dissonance. To resolve this tension, people might either conclude that minds are exempt from physical principles—that they *aren’t physical*, or possibly, that they are even outright *ethereal*.

Bloom’s proposal generates several testable predictions. First, it suggests that, as we watch agents engage in quotidian behavior, we ought to simultaneously activate the physics and mindreading systems; both predictions are borne out. Human bodies and objects engage a common frontoparietal brain network (e.g., Pramod et al., 2022; Schwettmann et al., 2019; Zbären et al., 2023), as well as the mindreading system, including the temporoparietal junction (e.g., Carrington & Bailey, 2009; Hyde et al., 2015; Jamali et al., 2021; Richardson & Saxe, 2020; Saxe & Kanwisher, 2003).

A second prediction is that these two systems ought to clash. The signature of this clash is indeed detectable both behaviorally (Berent, 2021a; Sandoboe & Berent, 2021) and neutrally—in the anti-correlation observed between the physical and mindreading brain systems (Jack et al., 2013).

Finally, if Dualism arises from the clash between intuitive physics and mindreading, people with a weaker mindreading system should show a weaker clash, hence, weaker Dualism. Past research has shown that autistic individuals show weaker mindreading than neurotypicals (e.g., Atherton & Cross, 2019; Baron-Cohen et al., 1985, 2015; Bellesi et al., 2018; Buon et al., 2013; Frith, 2004; Jameel et al., 2019; Moran et al., 2011; Rogé & Mullet, 2011; Senju et al., 2009; Thiébaut et al., 2016; Zalla et al., 2009), and that, within the neurotypical population, males show weaker mindreading than females (e.g., Adenzato et al., 2017; Baron-Cohen et al., 2001; Dorris et al., 2022; Groen et al., 2015; Hünefeldt et al., 2021; Khorashad et al., 2015; Kirkland et al., 2013; Quesque et al., 2022; Vellante et al., 2013). Thus, autistic people and males ought to show weaker Dualism than neurotypicals, and neurotypical males should show weaker Dualism than females. These predictions were borne out; moreover, the magnitude of Dualism correlated with mindreading abilities (Berent, 2023; Berent et al., 2022).

As noted, whether Dualism arises from core knowledge will not be addressed here. Nonetheless, the natural origins of Dualism can shed light on our two questions of interest: (a) how people conceive of bodies and minds; and (b) do they contrast bodies and minds implicitly.

### Properties of Minds and Bodies

If Dualism arises naturally, from core knowledge, then core knowledge could shape our intuitions about what bodies and minds *are*. Generally speaking, bodies ought to manifest the physical properties of objects.

Past research has shown that people—both adults and infants—intuitively expect objects to be *bounded* and *solid* (Spelke, 2022). Young infants know that a solid screen cannot pass through a solid box (Baillargeon, 1987). They can likewise count the number of bounded, solid objects, but fail to count stuff (e.g., sand; Huntley-Fenner et al., 2002) or objects that break in two (Cheries et al., 2008; violating boundedness).

The emergence of this knowledge in young infants (Baillargeon, 1987; Valenza et al., 2006) and in nonhuman animals (Chiandetti & Vallortigara, 2011; Regolin & Vallortigara, 1995) suggests that the principles of boundedness and solidity constitute core knowledge of objects (Spelke, 2022). Moreover, these principles continue to constrain cognition throughout life. Adults, for instance, can better track objects that move in continuous trajectories compared to ones that split (violating “boundedness”; Mitroff et al., 2004) or seem to “pour” (violating “solidity”; vanMarle & Scholl, 2003).

What properties people might ascribe to minds is less certain. One possibility is that people merely consider minds as non-physical. If so, they ought to simply be *less* likely to associate minds with the core properties of objects (e.g., solidity, having insides). On a stronger hypothesis, people might squarely view minds as *opposite* to bodies—as outright ethereal. Here, we test these intuitions.

We should note that the predicted attributes of objects and minds are strictly *relative*: we expect people to be *more likely* to associate bodies with physical properties (more than with nonphysical properties), and we expect the converse for minds. This, however, is not to say that minds would *never* be assigned any embodied physical attributes.

This prediction doesn’t follow because Dualism is unlikely to “play solo”—it is not the only bias to drive human cognition, and other competing biases might suggest just the contrary. For instance, there is evidence that people are also intuitive essentialists (e.g., Dar-Nimrod et al., 2014; Gelman & Wellman, 1991), and per Essentialism, psychological traits that define one’s innate essence are *embodied* (Berent, 2021c). So, whether in any given instance, a psychological trait would appear as disembodied (per Dualism) or embodied (per Essentialism) ought to depend on the seesaw balance between these competing, violable forces (Berent, 2021b).

For this reason, our predictions are strictly relative: *all things being equal*, bodies ought to be more strongly associated with physical properties than nonphysical properties, whereas for minds, this should not be the case (per the weaker hypothesis) or could possibly be the converse (per the strong view).

### Dualist Intuitions May Be Implicit

If Dualism is rooted in human cognition, then it would be easy for this Dualist bias to slip by, resulting in reasoning errors even in individuals who hold explicit beliefs to the contrary. As a result, Dualist intuitions could apply tacitly and implicitly.

Whether they do is uncertain. To date, the evidence for Dualism has come from procedures that elicit deliberate, off-line decisions (e.g., about the afterlife; Barrett et al., 2021; Bering & Bjorklund, 2004; body replication: Hood et al., 2012). While such judgments are demonstrably modulated by priming (e.g., Berent & Platt, 2021b; Forstmann & Burgmer, 2015), both the priming manipulation and the experimental probes involved explicit statements.

The Brief Implicit Association task (BIAT) could shed light on this question. The Implicit Association Task (Greenwald & Banaji, 2017; Kurdi et al., 2019) and its variant, the Brief Implicit Association Task (BIAT; Nosek et al., 2005, 2014) present the gold-standard for implicit processing. If Dualism is a tacit bias, then evidence for Dualism should emerge in the BIAT. Moreover, implicit Dualism could emerge even in individuals who explicitly negate Dualism.

Past research (e.g., Nosek et al., 2005, 2014) has used the BIAT to examine whether participants tacitly associate certain social categories (e.g., democrat) and attributes (e.g., good). To this end, each trial presented a target word (e.g., Obama) along with a category-attribute pair (e.g., Democrats/Good); the task was to quickly determine whether the target word belongs to one category/attribute or both: Democrat and Good targets elicit “yes” responses whereas those that aren’t Democrat or aren’t Good elicit “No.”

If the category-attribute association is implicit, then category-attribute pairings that are congruent with this belief ought to facilitate responses. For example, democrat participants ought to respond more readily to Democrats/Good, whereas republicans should show the converse. This is precisely what the results suggest (Nosek et al., 2014). Here, we deploy the BIAT to gauge intuitions about minds and bodies.

## THE PRESENT EXPERIMENTS

To determine whether participants are implicit Dualists, here, we asked participants to classify target words into one of two categories—body or mind. Each such category, in turn, was paired with one of two attributes, depicting either a physical property of bodies or their converse. Of interest is whether the physical attributes are associated *only* with bodies, but not minds.

To explore the role of boundedness, Experiments 1–2 contrasted the attributes object (which is bounded) and stuff (which isn’t). Experiments 3–5 contrasted solid and airy—a test of solidity. Each such attribute paired with the categories mind and body.

Since bodies exhibit the physical properties of objects, we expect participants to respond to the body category more readily (i.e., better response sensitivity, *d*′, and faster RT) when it is associated with physical properties (i.e., object, solid) than their converse (i.e., stuff, airy). The critical question concerns responses to mind words.

We consider two hypotheses. The weaker one states that minds merely seem *distinct* from physical objects. If so, physical properties should not facilitate responses to the mind. A stronger hypothesis states that minds are further considered as ethereal, i.e., as decidedly *not* physical. If so, physical properties should implicitly *impede* responses to mind targets. Experiments 1–3 explore these hypotheses. Experiments 4–5 further evaluated whether people who explicitly endorse Physicalism could still be closet Dualists.

## EXPERIMENT 1

In Experiment 1, the mind category corresponded to epistemic states (knowledge/beliefs) and it was contrasted with body (e.g., hand); attributes were object or stuff (e.g., chair vs. water).

Since bodies and objects are both bounded, whereas stuff isn’t, the body-object pairing should seem congruent, it should facilitate response relative to body-stuff. Critically, if minds seem distinct from bodies, then minds may not appear bounded. Consequently, object should selectively facilitate responses *only* to body, but not to mind.

### Methods

#### Participants.

Participants (*N* = 32) were University undergraduate students, native English speakers. They took part in this experiment in partial fulfillment of course requirements.

Sample size was informed by pilot work (based on Experiment 4; *N* = 32), in which the predicted interaction yielded a *η*_p_^2^ = .557 (in dprime) and *η*_p_^2^ = 0.62 (in RT). A power calculation suggested that a sample size of merely four participants is likely to yield the predicted interaction at a power of .8 and the alpha level of .05. Accordingly, in all subsequent experiments, we arbitrarily set the minimal sample size to *N* = 32. All procedures were reviewed and approved by the Institutional Review Board at the local institution; all participants signed informed consent forms.

#### Materials and Design.

The 16 target words represented the categories body (hand, leg, fingers, toes) and mind (knowledge, belief, ideas, thoughts), and the attributes object (chair, table, ball, pen) and stuff (water, sand, snow, mud).

Experimental design and procedure closely followed past BIAT research (Nosek et al., 2014). Each experiment consisted of eight blocks of experimental trials. Each such block featured all 16 experimental targets along with a category-attribute pair (e.g., body-object). Within a block, half of the targets belonged to either the featured category or the featured attribute—these are the focal targets; the other half were non-focal targets, as they did not belong to either the category or attribute.

Each participant was presented with all four 2 category × 2 attribute pairings, and each such pairing (e.g., body-object) was featured in a separate block of trials, which was repeated twice, for a total of 128 experimental trials (4 blocks × 2 repetitions × 16 targets). Block order was counterbalanced (for details, see Supplementary Materials). To master the task, participants were given multiple sets of practice sections, detailed in the Supplementary Materials.

#### Procedure.

Participants were tested in groups of 1–3 participants. After going through several practice phases (for details, see Supplementary Materials), participants were administered the experimental task. Participants initiated each block by pressing a designated letter key. Their response triggered the presentation of a fixation (+), displayed at the center of the screen for 1 second (see Figure S1). The fixation was immediately followed by a single target word (centered) along with the focal category (e.g., body) and an attribute (e.g., object), centered at the top of the screen. Participants were asked to determine whether the target belonged to the category or attribute; if it belonged to either, participants were asked to press ‘p’; if it didn’t belong to either, they were asked to press ‘q.’ Their responses immediately triggered the next trial. There was no feedback in the experimental trials. The data from all experiments is provided as a Supplementary material file.

### Results and Discussion

**Figure 1.**
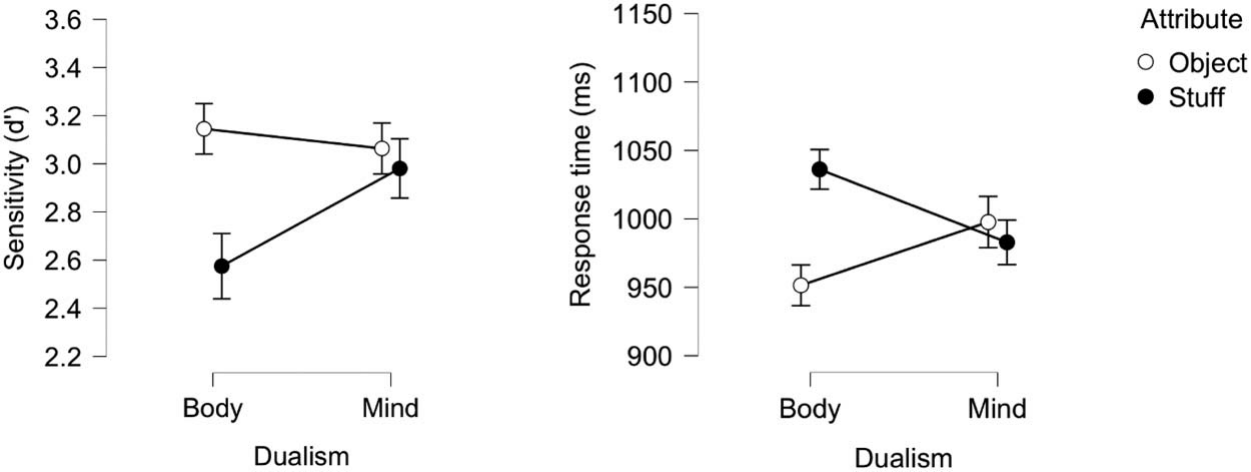
Mind-Body responses as a function of Attribute (object vs. stuff) in Experiment 1.

Figure 1 depicts the effect of the target category (body vs. mind) and attribute (object vs. stuff) on sensitivity (dprime; hit = correct “yes” response to focal targets; false alarms are erroneous “yes” response to nonfocal targets) and correct responses time. In this and all subsequent experiments, the response time (RT) analyses excluded outliers, defined as correct responses falling 2.5 *SD* above the mean or faster than 200 ms (less than 4% of the total observations). All analyses were conducted in JASP (JASP Team, 2022) and (unless otherwise noted) all figures were generated therein, whereby SE are normalized standard errors (Morey, 2008).

An inspection of the means suggests that participants were more sensitive and faster to categorize targets as body when the body-category was paired with object relative to stuff. This, however, was not the case for mind.

A 2 Dualism (body/mind) × Attribute (object/stuff) ANOVA yielded a significant effect of Attribute (In RT: *F*(1, 31) = 4.73, *p* = .04, *η*_p_^2^ = .132; In sensitivity: *F*(1, 31) = 4.90, *p* = .03, *η*_p_^2^ = 0.137), as, overall, the object attribute produced faster and more sensitive responses than stuff. The main effect of Dualism was not significant (In RT: *F* < 1; in sensitivity: *F*(1, 31) = 3.22, *p* > .08, *η*_p_^2^ = .094). Critically, the Dualism × Attribute interaction was significant in both RT (*F*(1, 31) = 10.27, *p* < .003, *η*_p_^2^ = .249) and sensitivity (*F*(1, 31) = 4.97, *p* = .03, *η*_p_^2^ = .138).

We next evaluated the hypothesized simple main effect of attribute. Results showed that responses to the body category were faster (*F*(1, 31) = 19.84, *p* < .001) and more sensitive (*F*(1, 31) = 9.02, *p* < .005) when body was paired with object than with stuff. This, however, was not the case for the mind category, as responses for object and stuff didn’t differ (*F* < 1, in both sensitivity and RT).

To determine whether this null effect of attribute (for mind) indicates evidence *for* the null hypothesis (as opposed to absence of evidence), we next submitted the results to a Bayesian Factor analysis (conducted in JASP). For mind, the support for the null hypothesis was moderate (in sensitivity: BF_0_ = 7.304; in RT: BF_0_ = 3.168). For body, by contrast, we found strong support for the *alternative* hypothesis (that object facilitates response relative to stuff) in sensitivity (BF_1_ = 15.234); in RT, the support was extreme (BF_1_ = 503.612).

These results suggest that bodies are more strongly associated with bounded objects than stuff; this is in line with the hypothesis that bodies are perceived as bounded entities. Critically, people do not seem to project boundedness to minds. Thus, people do not consider minds akin to physical bodies, in line with the weaker hypothesis above.

## EXPERIMENT 2

Experiment 2 explored the role of boundedness in another mental state—emotions. Unlike epistemic states (in Experiment 1), emotions seem partly embodied (Berent et al., 2020). Still, when compared to bodily organs (e.g., leg), emotions should still appear less physical. If so, object should only facilitate response to body, not mind.

### Methods

Sample size (*N* = 32) and participants characteristics were as in Experiment 1.

The mind category featured four emotions (fear, surprise, anger, happiness); the body targets were as in Experiment 1, except that plural nouns (toes, fingers) were replaced with singulars (toe, finger). object-stuff attributes (chair, table, ball, pen; water, sand, snow, mud) were as in Experiment 1.

### Results and Discussion

**Figure 2.**
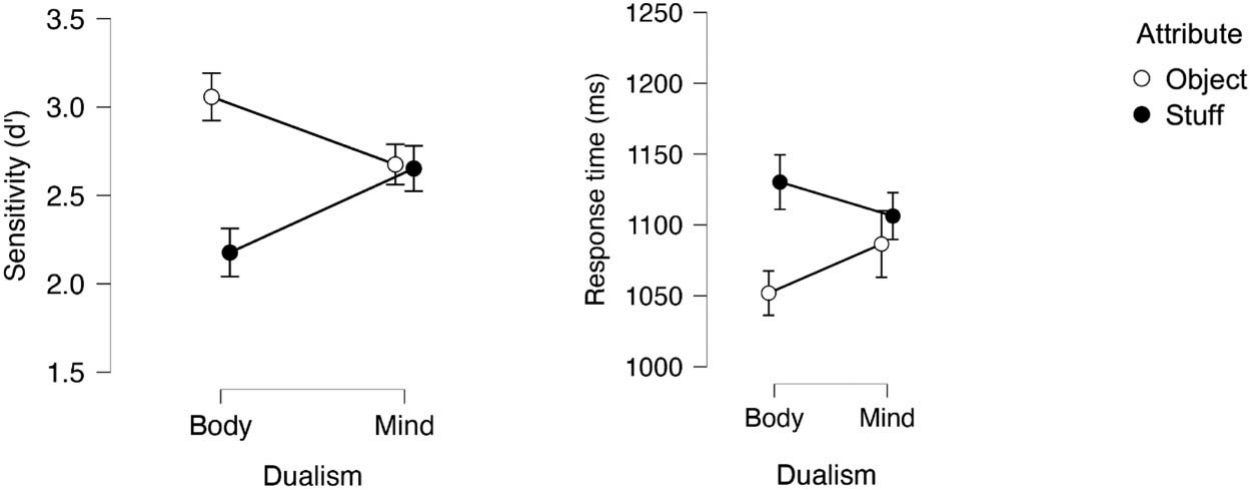
Mind-Body responses as a function of Attribute (object vs. stuff) in Experiment 2.

An inspection of the means (Figure 2) suggests that, as in Experiment 1, responses to the body category were more sensitive and faster when body was paired with object (than with stuff). This, however, was not the case for mind.

A 2 Dualism × 2 Attribute ANOVA yielded a reliable main effect of Attribute, as the object attribute yielded faster (*F*(1, 31) = 5.12, *p* = .03, *η*_p_^2^ = .142) and more sensitive (*F*(1, 31) = 8.72, *p* < .006^2^, *η*_p_^2^ = .106) responses than stuff. The main effect of Dualism was not significant (*F* < 1, in both speed and sensitivity). The interaction, however, was highly significant in the analysis of sensitivity (*F*(1, 31) = 17.82, *p* < .001, *η*_p_^2^ = .096; in RT (*F*(1, 31) = 3.41, *p* = .07, *η*_p_^2^ = .099).

We next probed for the hypothesized simple main effect of attribute. As predicted, when body was paired with object, responses were more sensitive (*F*(1, 31) = 19.89, *p* < .001) and faster (*F*(1, 31) = 10.82, *p* < .003) compared to the body-stuff pairing. This, however, was not the case for mind (*F* < 1, in both sensitivity and RT).

A Bayesian Factor analysis showed that, for mind, there was moderate support for the null hypothesis (in sensitivity: BF_0_ = 5.856; in RT: BF_0_ = 8.215). For body, by contrast, the support for the alternative hypothesis (that object facilitates response relative to stuff) was strong, in RT (RT: BF_1_ = 28.96) and extreme, in sensitivity (BF_1_ = 510.854).

These results confirm that people associate body with object more strongly than with stuff, possibly, because bodies seem bounded. Critically, people do not exhibit the same association for mind, and this is the case regardless of whether mind is instantiated by epistemic states (in Experiment 1) or emotions (in Experiment 2).

## EXPERIMENT 3

Experiments 1–2 suggest that bodies are perceived as bounded. While this is *not* the case for minds, it is unclear whether participants further consider minds as ethereal.

Experiments 1–2 cannot address this question, as none has featured attributes that are unambiguously non-physical. Specifically, stuff (in Experiment 1–2) still conveys matter, but it does not explicitly imply “ethereal.” These attributes, then, may have only allowed us to evaluate the weaker hypothesis that minds *lack* the physical properties of bodies, but not the stronger hypothesis that minds are *not* physical, i.e., ethereal.

To test the stronger hypothesis, Experiment 3 featured the physical-ethereal contrast by comparing solid with airy. Solidity is seen as a physical property of objects by both human infants (Baillargeon, 1987) and chicks (Chiandetti & Vallortigara, 2011). airy, by contrast, is associated with the intangible and illusory, akin to the ethereal. We thus expected people to associate solid with the physical and airy with the ethereal.

Before we consider the results of Experiment 3, we first sought to validate our intuitions that its attributes explicitly convey a solid-ethereal contrast (unlike those in previous experiments). We next turn to the results of Experiment 3.

### Methods

Sample size (*N* = 32) and participants characteristics were as in Experiment 1.

Attributes were solid (chair, table, stone, brick) and airy (wind, fume, smoke, steam); the categories were body (hand, leg, finger, toe) and mind (knowledge, belief, idea, opinion). Due to a programming error, one of the four experimental lists replaced the target “brick” with “body”; these trials were filtered out of all analyses (see Supplementary Materials for further details).

### Rating Experiment

To evaluate our intuitions regarding the semantics of the attributes in Experiments 1–3, we first invited a group of Prolific workers (*N* = 20, native English speakers) to rate all six attributes. Participants judged each attribute (randomized) as either material or ethereal, using a five-point scale (1 = highly ethereal; 2 = ethereal; 3 = neither ethereal nor material; 4 = material; 5 = highly material).

To determine whether participants perceive an attribute as physical, we contrasted the mean against the scale’s neutral midpoint (3) using one-sampled *t* tests. To compare the relative materiality of matched attributes within each experiment, we further contrasted them using a paired sample *t* test. Table 1 presents the results.

**Table 1.** Rating results

(a) One-sample *t* tests (against 3)						
**Experiment**	**Attribute**	**Mean**	***SE***	***t* (20)**	***p***	**Cohen’s *d***
1–2	Object	4.76	0.12	14.98	<.001	3.27
	Stuff	4.19	0.26	4.86	<.001	1.06
3	Solid	4.33	0.22	7.68	<.001	1.68
	Airy	1.95	0.29	−5.22	<.001	−1.14
(b) Paired-samples *t* tests						
**Experiment**	**Attribute**			***t* (20)**	***p***	**Cohen’s *d***
1–2	object-stuff			3.23	0.004	0.705
3	solid-airy			9.07	<.001	1.979

As expected, the physical attributes (object, solid) were rated as material, significantly above the scale’s midpoint, as well as higher than their converse (stuff, airy). However, participants did not consider stuff as immaterial; in fact, they rated it as clearly material (i.e., *above* the neutral midpoint).

The rating results explain why stuff did not facilitate response to mind in Experiments 1–2. Since stuff does not convey the ethereal, it should not have been associated with mind, even if participants were staunch Dualists. airy, by contrast, was rated as clearly ethereal. If people indeed consider minds as ethereal, then in Experiment 3, airy should selectively facilitate response to mind, whereas solid should facilitate response to body.

### Results and Discussion (BIAT)

**Figure 3.**
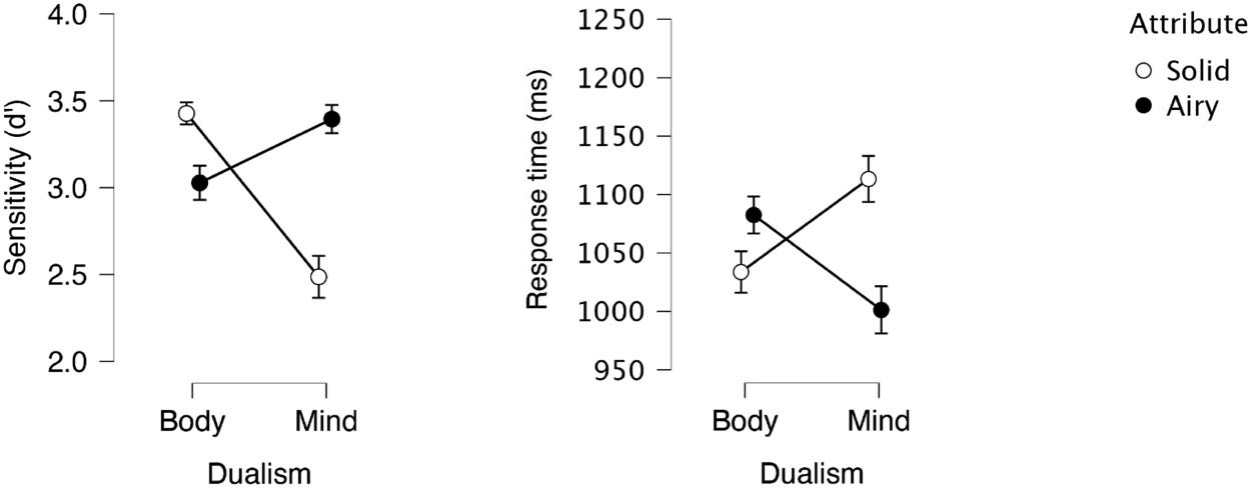
Mind-Body responses as a function of Attribute (solid vs. airy) in Experiment 3.

An inspection of the means (Figure 3) suggests that solidity had opposite effects on body and mind. For the body category, solid facilitated response (relative to airy). For mind, by contrast, responses were faster and more sensitive with airy.

The 2 Dualism × 2 Attribute ANOVA yielded a reliable main effect of Dualism in sensitivity (*F*(1, 31) = 9.01, *p* < .005, *η*_p_^2^ = .059; in RT: *F* < 1), as body produced better sensitivity than mind.

There was also a significant main effect of Attribute (In Sensitivity: *F*(1, 31) = 7.75, *p* < .009, *η*_p_^2^ = .047; In RT (*F*(1, 31) = 2.11, *p* < .16, *η*_p_^2^ = .064), as solid produced better sensitivity and faster responses than airy. Critically, the interaction was highly significant in both sensitivity (*F*(1, 31) = 49.57, *p* < .001, *η*_p_^2^ = .309) and RT (*F*(1, 31) = 17.98, *p* < .001, *η*_p_^2^ = .367).

We next probed the hypothesized simple main effects of Attribute. As expected, solid elicited better sensitivity (*F*(1, 31) = 14.04, *p* < .001; in RT: (*F*(1, 31) = 3.62, *p* < .07) to body (relative to airy). Critically, responses to mind showed the opposite pattern: airy yielded more sensitive (*F*(1, 31) = 36.53, *p* < .004) and faster (*F*(1, 31) = 12.47, *p* < .001) responses (relative to solid).

A Bayesian Factor analysis evaluated the alternative hypothesis that solid facilitates response to body (relative to airy). The support for this hypothesis was anecdotal in RT (BF_1_ = 1.796), but very strong in sensitivity (BF_1_ = 85.517). Critically, support for the hypothesis that airy facilitates response to mind (relative to solid) was very strong in RT (BF_1_ = 51.083) and extreme, in sensitivity (BF_1_ = 32,523). Thus, not only do participants selectively view bodies as physical, but they further view minds as squarely ethereal.

## EXPERIMENT 4

Having shown that Dualism applies implicitly (in Experiments 1–3), we next sought to determine whether implicit Dualism dissociates from explicit, declarative beliefs. Simply put, are participants closet Dualists despite explicit beliefs to the contrary?

To find out, Experiment 4 contrasted evidence from implicit and explicit measures of Dualism. The implicit measure was the BIAT experiment, conducted precisely as in Experiment 3. Explicit measures asked participants to indicate their agreement with statements concerning the mind-body link and the afterlife. If people are closet Dualist, then airy should selectively facilitate response to mind (but not body) even in individuals who explicitly endorse physicalism (i.e., the view of minds as indistinguishable from bodies).

### Methods

Because this experiment was concerned with individual differences, we arbitrarily increased the sample size to 60 participants. Their characteristics were as in Experiment 1. Data from one participant was lost due to a technical problem.

All participants first took part in a BIAT task, exactly as in Experiment 3. They were next given two explicit measures of Dualism (for the full materials, see Appendix II, Supplementary Materials): the mind-body task and the afterlife task.

The mind-body task (adapted from Stanovich, 1989) presented participants with 27 statements, expressing explicit claims about the mind-body link. 13 of these statements expressed Dualism (e.g., *The mind is not part of the brain but it affects the brain*) and 14 expressed Physicalism (*When I use the word “mind,” it is just a shorthand term for the complicated things that my brain does*).

The afterlife task (adapted from Bering, 2002) featured 19 statements concerning the afterlife—9 statements expressed beliefs in the afterlife (*In the premature death of someone close, some comfort can be found in knowing that in some way the deceased is still existing*) and 10 statements denied it (*Earthly existence is the only existence we have*).

In both tasks, responses were given using a 1–5 scale (1 = highly disagree; 5 = highly agree). Responses to the physicalists/afterlife-denying statements were reverse-coded, such that high values invariably endorse the afterlife in line with Dualism.

### Results and Discussion

#### Explicit Dualism.

**Figure 4.**
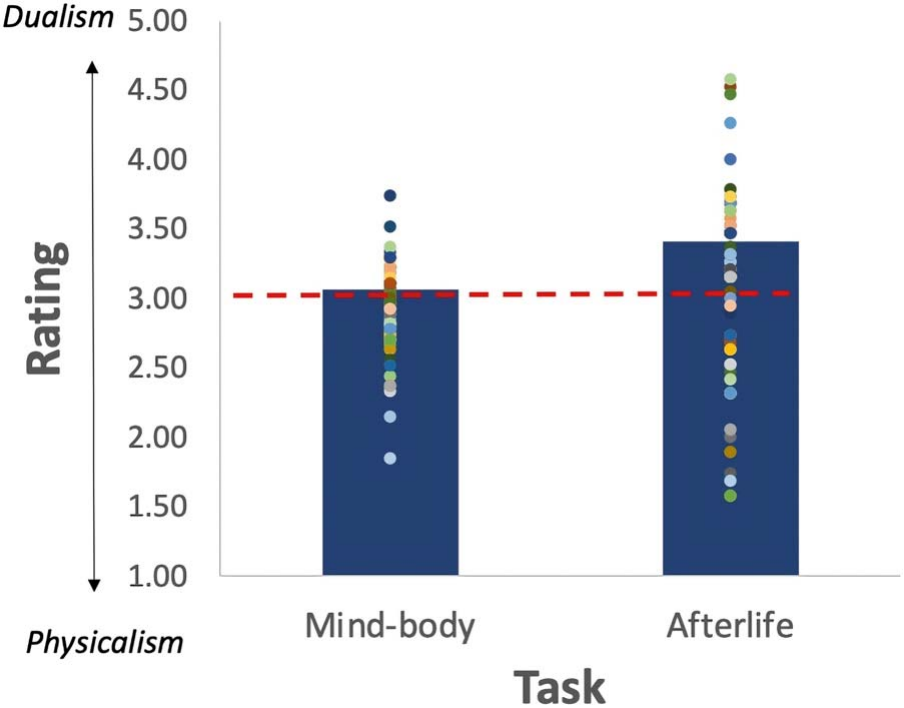
Mean rating in the explicit mind-body and afterlife tasks in Experiment 4. Dots capture the means of individual participants; the red line indicates the scale’s “neutral” midpoint.

An inspection of the means (Figure 4) showed no evidence for explicit Dualism, as the means hovered around the “neutral” midpoint of the scale (3). A one-sampled *t* test indicated that, in the afterlife task, responses did not significantly differ from the “neutral” midpoint (*M* = 3.08; *t* < 1). In the mind-body task, the mean was significantly lower than the neutral midpoint (*M* = 2.91, *t*(59) = −2.15, *p* = .04, Cohen *d* = −0.277). For the afterlife question, the support for the alternative Physicalist hypothesis was anecdotal, (BF_1_ = 0.08), whereas for the mind-body question, the support for Physicalism was very strong (BF_1_ = 47.87).

Thus, when Dualism was evaluated explicitly, participants’ attitudes were either neutral (in the afterlife task) or squarely Physicalist (in the mind-body task). Of interest is whether these “outwards physicalist” participants nonetheless show implicit evidence for Dualism. To find out, we next examine their performance on the BIAT tasks.

#### BIAT Results (All Participants).

**Figure 5.**
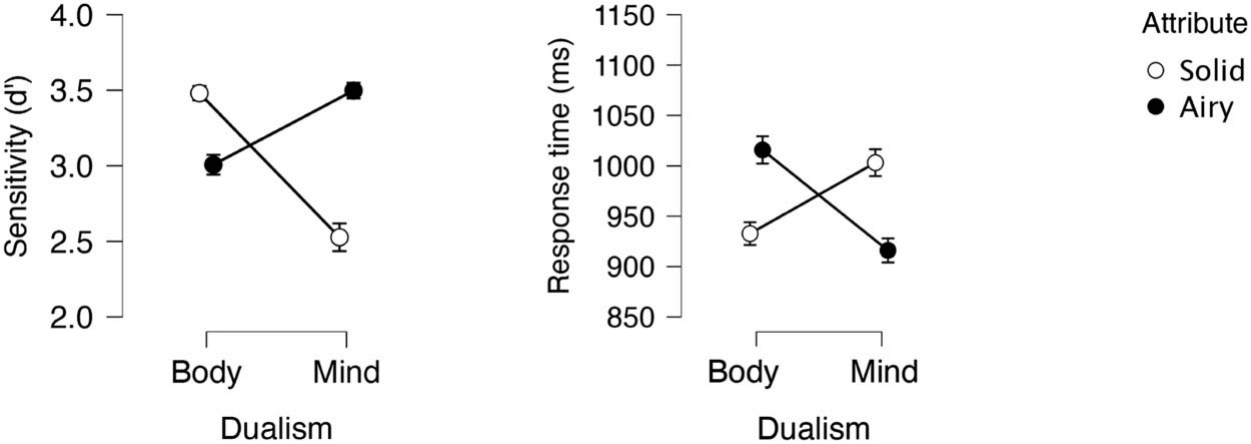
Mind-Body responses as a function of Attribute (solid vs. airy) in Experiment 4.

An inspection of the BIAT means (Figure 5) suggests that, as in Experiment 3, solidity selectively facilitated responses to body, but impaired responses to mind.

A 2 Dualism × 2 Attribute ANOVA yielded reliable main effects of Attribute (In sensitivity: *F*(1, 59) = 11.79, *p* < .001, *η*_p_^2^ = 0.167; in RT: *F* < 1) and Dualism (In sensitivity: *F*(1, 59) = 14.21, *p* < .001, *η*_p_^2^ = 0.194; in RT: *F*(1, 59) = 1.91, *p* > .17, *η*_p_^2^ = 0.031), as airy elicited better sensitivity than solid, and body elicited better sensitivity than mind. Critically, the interaction was highly significant in both sensitivity (*F*(1, 59) = 123.72, *p* < .001, *η*_p_^2^ = 0.677) and RT (*F*(1, 59) = 71.24, *p* < .001, *η*_p_^2^ = 0.547).

A simple main effect analysis showed that responses to body were faster (*F*(1, 59) = 19.48, *p* < .001) and more sensitive (*F*(1, 59) = 37.09, *p* < .001) with solid relative to airy. Critically, responses to mind showed the opposite, as here, sensitivity (*F*(1, 59) = 73.21, *p* < .001) and speed (*F*(1, 59) = 20.51, *p* < .001) were better with airy.

The support for the alternative hypothesis was extreme for both body (in sensitivity: BF_1_ = 299,905; in RT: BF_1_ = 930) and mind (In sensitivity: BF_1_ = 2,820,000,000; in RT: BF_1_ = 1,347). These results confirm that, despite their explicit endorsement of Physicalism, participants showed implicit Dualism.

#### Physicalist Participants.

Could the dissociation between explicit and implicit Dualism arise from different subsets of participants? To counter this concern, we next reanalyzed the BIAT results after excluding from the analysis all participants who explicitly endorsed Dualism. Thus, we only included participants whose mean responses in the mind-body and afterlife tasks was lower than 3. Sixteen participants met this criterion.

As expected, the mean response in the mind-body (*M* = 2.65, *t*(15) = −4.89, *p* < .001, *d* = −1.224) and afterlife (*M* = 2.28, *t*(15) = −6.76, *p* < .001, *d* = −1.683) tasks were both significantly below the neutral midpoint, confirming that indeed, these participants were staunch explicit Physicalists. The support for the Physicalist hypothesis was extreme (for body-mind: BF_1_ = 317, for afterlife: BF_1_ = 6,330).

**Figure 6.**
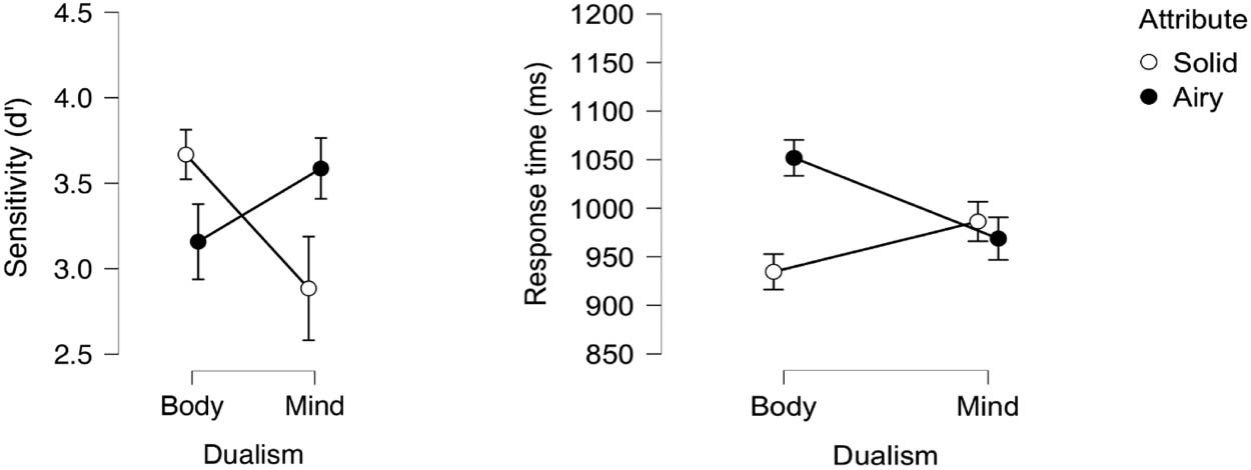
Mind-Body responses for Physicalist participants in Experiment 4.

Implicitly, however, these participants showed evidence for Dualism (Figure 6). The 2 Dualism × 2 Attribute interaction was significant in both sensitivity (*F*(1, 15) = 24.38, *p* < .001, *η*_p_^2^ = 0.662) and Response time (*F*(1, 15) = 37.73, *p* < .001, *η*_p_^2^ = 0.716).

The interaction emerged because responses to body were more sensitive (*F*(1, 15) = 16.45, *p* < .005) and faster (*F*(1, 15) = 22.99, *p* < .001) when body was paired with solid relative to airy. Critically, responses to mind were more sensitive when mind was paired with airy (relative to body: *F*(1, 15) = 14.62, *p* < .002; or RT, these attributes did not differ, *F* < 1).

For body, the support for the alternative hypothesis was extreme (in sensitivity: BF_1_ = 275.68; in RT: BF_1_ = 268.00). For mind, the data offered moderate evidence for the alternative hypothesis in sensitivity (BF_1_ = 8.83); in RT, there was anecdotal evidence for the null hypothesis (BF_1_ = 0.42). These results make it clear that these staunch Physicalists were closet Dualists. Altogether, then, Experiment 4 shows that Dualism is a tacit belief that can dissociate from explicit Dualism. Experiment 5 next replicated these results using a pre-registered plan (another such pre-registered replication is reported in the Supplementary Materials).

## EXPERIMENT 5

### Methods

Experiment 5 was preregistered (https://osf.io/mxw94/?view_only=ca77820f2f5d4a978efa48ae56d74cc8); it was a near-exact replication of Experiment 4, with the following differences. First, the sample size was set to 80 participants; of these, 66 were Northeastern students and the remaining 14 were Prolific workers. Second, participants took the study online (as opposed to in person). To this end, we implemented the BIAT on the PsychExp platform. Because its trial structure is canned to require a forced choice between two options, we further slightly modified the trial structure. Thus, in Experiment 5, each trial presented two options. One was the category/attribute combination (e.g., solid, body); the other was “neither” (see Figure S2). Participants were instructed to press “q” if the target belongs to the category/attribute combination; otherwise, choose “neither” (by pressing “p”).

### Results and Discussion

#### Explicit Dualism.

**Figure 7.**
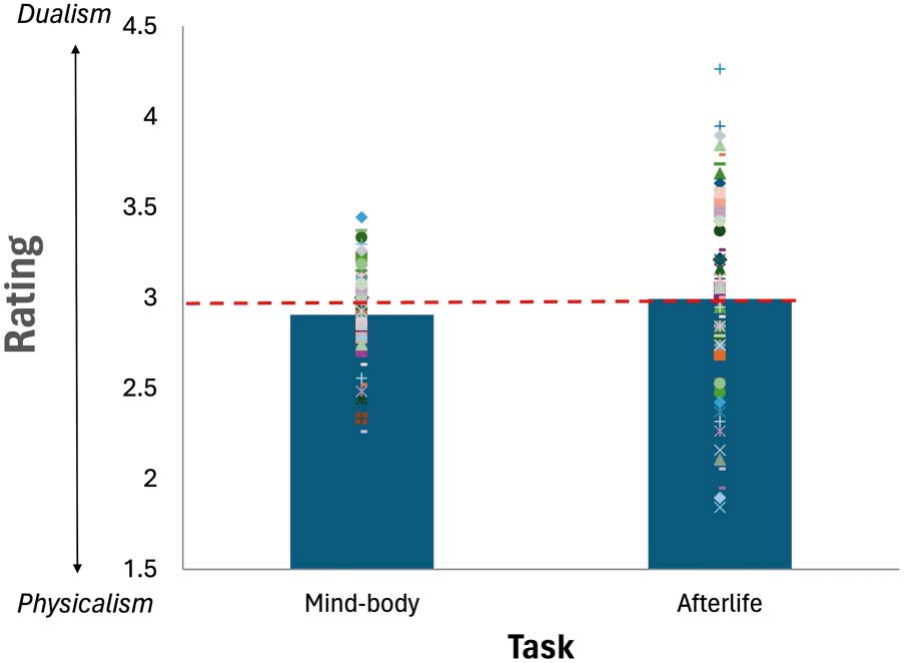
Mean rating in the explicit mind-body and afterlife tasks in Experiment 5. Dots capture the means of individual participants; the red line indicates the scale’s “neutral” midpoint.

When considering the entire sample (*N* = 80), participants showed no explicit evidence for Dualism (Figure 7). Their endorsement of the afterlife hovered around the neutral scale midpoint (*M* = 2.99, *t*(79) < 1), and they outright rejected that the mind is distinct from the body (i.e., below, the neutral midpoint: *M* = 2.91, *t*(79) = 3.41, *p* < .001).

A Bayesian factor analysis showed that the support for the Physicalist hypothesis in the mind-body probe was very strong (BF_1_ = 47.87); in the afterlife condition, there was very strong support for the null hypothesis (BF_1_ = 0.132). Thus, when asked explicitly about their afterlife beliefs, participants offered no evidence for Dualism, and when asked to identify the mind-body links, they outright endorsed Physicalism.

#### Implicit Dualism.

**Figure 8.**
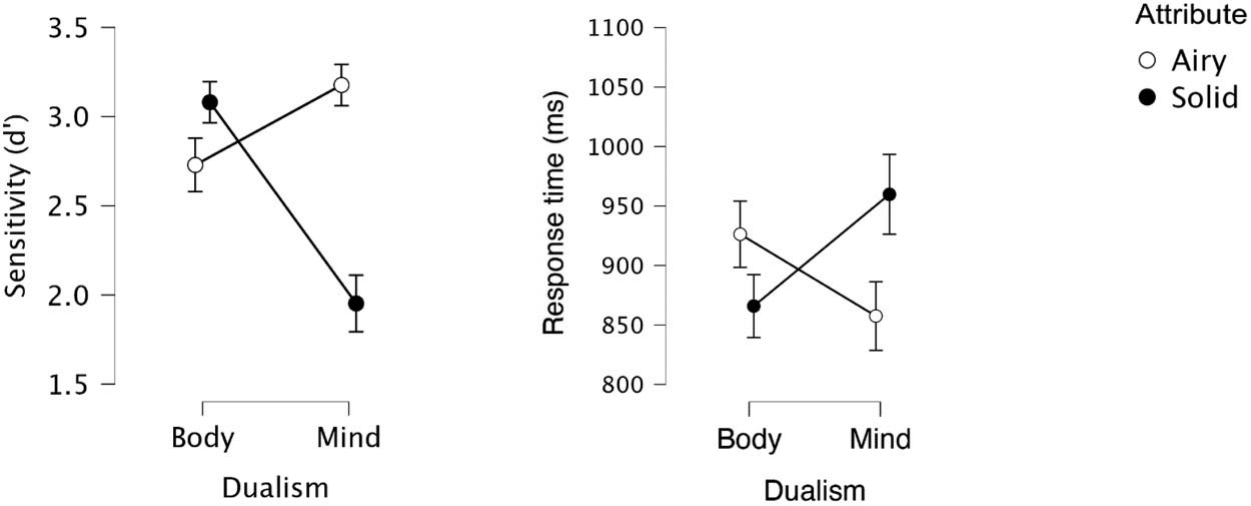
Mind-Body responses in Experiment 5.

Results from the BIAT, however, offered evidence for implicit Dualism (see Figure 8). The 2 Dualism × 2 Attribute interaction was significant in both sensitivity (*F*(1, 79) = 149.42, *p* < .001, *η*_p_^2^ = 0.654) and response time (*F*(1, 78) = 29.16, *p* < .001, *η*_p_^2^ = 0.272). The interaction emerged because responses to body were more sensitive (*F*(1, 79) = 14.24, *p* < .001) and faster (*F*(1, 78) = 9.47, *p* < .001) when body was paired with solid relative to airy. Critically, responses to mind were more sensitive when mind was paired with airy (relative to body), and this was the case in both sensitivity (*F*(1, 79) = 153.89, *p* < .001) and response time (*F*(1, 78) = 18.36, *p* < .001).

For body, the sensitivity analysis offered extreme support for the alternative hypothesis (BF_1_ = 140.82; in RT: BF_1_ = 0.02). Critically, for mind, there was extreme support for the alternative hypothesis in both sensitivity (BF_1_ = 391,700,000,000,000,000) and in response time: BF_1_ = 16,416).

#### Physicalist Participants.

As in Experiment 4, we next moved to identify a subgroup of Physicalist participants—those whose support of the afterlife and the mind-body divide were both lower than the scale’s neutral point (3). For these 28 participants, the mean response in the explicit Dualism tasks was significantly below the neutral midpoint for both the afterlife (*M* = 2.58, *t*(27) = 6.29, *p* < .001) and mind-body (*M* = 2.77, *t*(27) = 6.30, *p* < .001) questions. The support for the alternative hypotheses was extreme (for the afterlife: BF_1_ = 36,052; for the mind-body question: BF_1_ = 37,048). Thus, these participants explicitly rejected the persistence of the mind in the afterlife and its separation from the body.

**Figure 9.**
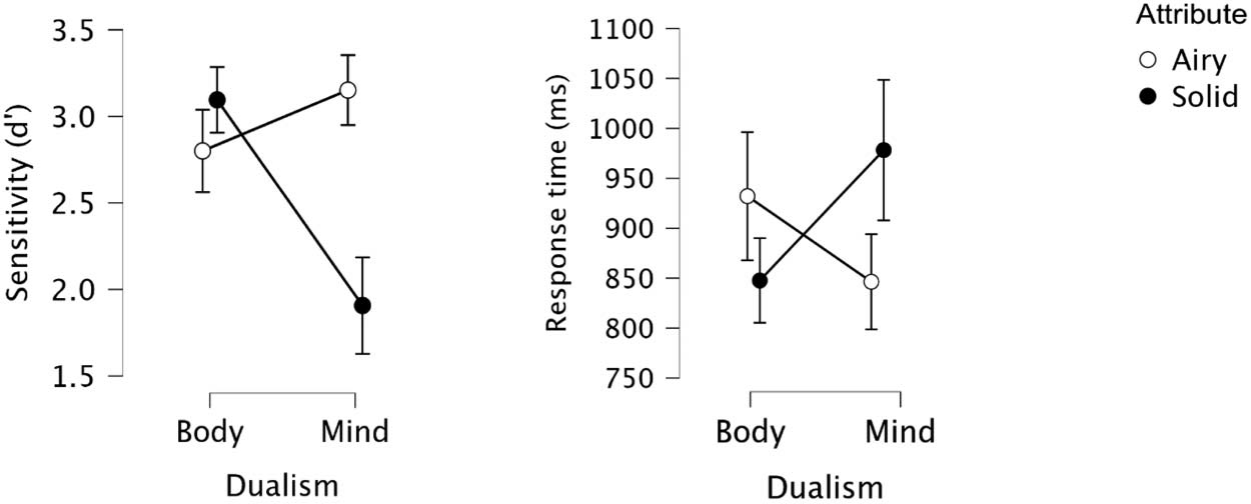
Mind-Body responses for Physicalist participants in Experiment 5.

Still, when probed in the BIAT, their responses showed evidence of Dualism (Figure 9). The Dualism × Attribute interaction was significant in both sensitivity (*F*(1, 27) = 58.03, *p* < .001, *η*_p_^2^ = 0.682) and response time (*F*(1, 27) = 15.13, *p* < .001, *η*_p_^2^ = 0.368). For body, responses were significantly faster with solid relative to airy (*F*(1, 27) = 6.06, *p* = .02); in sensitivity, this trend was not significant (*F*(1, 27) = 3.80, *p* = .06). Critically, responses to mind were significantly more sensitive (*F*(1, 27) = 48.16, *p* < .001) and faster with (*F*(1, 27) = 10.63, *p* < .01) AIRY (relative to solid).

The sensitivity analysis yielded anecdotal evidence for the alternative hypothesis for body (BF_1_ = 2.00) and extreme support for mind (BF_1_ = 169,345); in response time, there was strong support for the alternative hypothesis for both body (BF_1_ = 16.06) and mind (BF_1_ = 26.46). These results confirm that participants can tacitly contrast bodies and minds despite explicit beliefs to the contrary.

## GENERAL DISCUSSION

Do people tacitly believe that the mind is ethereal, distinct from the body? And how do they implicitly view bodies and minds?

Here, we explored the hypothesis that bodies are tacitly viewed akin to solid physical objects; minds; by contrast, seem either as not physical (i.e., as neither akin to objects or solid) or, on a stronger view, as the opposite—possibly, as stuff, or even ethereal.

To test this hypothesis, we had people classify target words as either body or mind; each such category, in turn, was paired with an attribute that either aligns with the putative properties of objects (i.e., object or solid) or its opposite (stuff, airy).

Experiments 1–5 showed that responses to body targets were systematically facilitated by physical properties—by object (relative to stuff; in Experiments 1–2), and solid (relative to airy, in Experiments 3–5). Critically, these physical attributes did not facilitate responses to mind (a conclusion supported by Bayesian Factor analysis). Moreover, when mind was paired with airy—an attribute that explicitly invokes the ethereal (in Experiments 3–5), now, responses were significantly facilitated (relative to solid); Experiment 6 (in the Supplementary Materials) offers yet another replication of this effect. Thus, not only do people consider the mind as distinct from the physical body, but they further view it as ethereal. Remarkably, this was the case even in participants who explicitly endorsed Physicalism (in Experiments 4–5).

These results, then, show for the first time that Dualism is an implicit belief that can be maintained despite explicit, conscious beliefs to the contrary—a notion that is in line with what the philosopher Tamar Gendler defines as *alief*—a mental state associated with a cluster of representational contents (here, about bodies and minds) that may be activated regardless of awareness, despite holding beliefs to the contrary. For instance, rubber vomit elicits disgust even if you believe it’s not real (Gendler, 2008). By the same token, implicit Dualism may lead one to view minds as ethereal, distinct from the body, even in participants who explicitly claim that bodies and minds are one and the same.

Why do participants tacitly contrast bodies and minds? And why do people believe that minds are ethereal, distinct from solid bodies? To be clear, our present results do not directly speak to this question. Nonetheless, both facts could be readily explained by the hypothesis that Dualism is anchored in a dissonance between two systems of core knowledge—intuitive physics, on the one hand, and mindreading, on the other (Bloom, 2004).

This proposal can explain why people define bodies and minds in *these* precise terms; for example, why people believe that bodies are solid (whereas minds aren’t). This belief can be traced to a system of “core knowledge” (Spelke, 2022), evident in newborn infants (Mascalzoni et al., 2013) and newly hatched chicks (Regolin & Vallortigara, 1995), which continues to play a role among adults (vanMarle & Scholl, 2003). If our intuitive understanding of the body is anchored in core knowledge, and if minds are distinct from bodies, then only bodies ought to seem solid (and not minds), just as our results show.

Moreover, the anchoring of Dualism in core knowledge explains why a Dualist belief can “slip by,” even in participants who explicitly believe that “the mind is just what the brain does,” as evident in the Dualist tendencies of people who vehemently deny Dualism.

Implicit biases are difficult to detect and control precisely because they operate rapidly and automatically (Morewedge & Kahneman, 2010). The implicit nature of Dualism thus explains why it is so pervasive. Indeed, its putative effects range from questions of pre-life (Emmons & Kelemen, 2014) to death (Bering & Bjorklund, 2004), with implications to neuroscience (Sandoboe & Berent, 2021; Weisberg et al., 2008), criminal justice (Aspinwall et al., 2012), and mental health (Ahn et al., 2017; Berent & Platt, 2021a).

The tension between implicit Dualism and explicit Dualist beliefs can further explain why the evidence for Dualism is contradictory—why Dualism is early-emerging and pervasive across cultures (Barlev & Shtulman, 2021; Bloom, 2004; Chudek et al., 2018), yet (when probed explicitly), participants sometimes deny that minds are fully ethereal (Barlev & Shtulman, 2021) and that minds can outlive bodies (Barrett et al., 2021).

At a broader level, the implicit nature of Dualism sheds light on why this bias derails reasoning about science, health and technology even among educated participants (Berent, 2021b). We recognize that some readers might object to our discussion of the “dangers of Dualism.” Indeed, Dualism remains a topic of lively debate in philosophy; the controversy regarding the “hard problem of consciousness” presents a notable example (e.g., Chalmers, 1996; Cogitate Consortium et al., 2025). If some trained philosophers endorse Dualism, then how could we accuse laypeople of a Dualist *bias*?

Let us clarify the debate and our assertions in this regard. When philosophers debate the physical correlates of consciousness, no one doubts that consciousness is *linked* to the brain; what’s at stake is whether consciousness can be *reduced* to brain states (Chalmers, 2000). This philosophical position, then, differs from the intuitive belief that the mind is ethereal, distinct from the physical body. Our concern is with intuitive Dualism and with the profound errors it promotes in laypeople’s *reasoning* about cognitive questions.

That such errors exist is a demonstrably fact. For example, it is well known that people prefer brain-based explanations to cognitive explanations, even when brain explanations are logically circular (Weisberg et al., 2008). All sides must agree that the endorsement of logical circularity is indefensible. Recent results have shown that this bias arises from Dualism (Sandoboe & Berent, 2021). And as such, Dualism evidently derails reasoning.

Dualism further drives the false presumption that, if a psychiatric disorder “shows up” in a brain scan, then the disorder in question is inborn (Berent & Platt, 2021a, 2021b). This logic is demonstrably flawed, as learning has a clear impact on the brain. Accordingly, the fact that a trait “shows up” on a brain scan provides no reliable indicator of whether it’s learned or innate. Dualism (along with Essentialism) suggests otherwise (Berent, 2021b).

Finally, Dualism has been linked to the false belief that, if an emotion “shows up” in the face, then that emotion is innate (Berent et al., 2020). Thoughts, on the other hand, seem disembodied, so people assume that thoughts can only arise by learning from experience (Berent, 2020; Berent et al., 2019; Wang & Feigenson, 2019).

What’s disturbing about these intuitions isn’t the *conclusions* they support. Questions of innateness remain widely contested in affective and cognitive science (e.g., Barrett, 2017; Lorenzi et al., 2025), so the conclusions advocated by laypeople could well turn out to be correct. Our concern is not with the conclusions but with the *logic* that supports them. Intuitive Dualism cannot support reliable inferences about the workings of the mind.

Our present results, however, demonstrate that intuitive Dualism applies tacitly and rapidly. And if implicit Dualism can take hold of educated laypeople, scholars may not be immune to its danger (e.g., Carruthers, 2020; Maggio et al., 2024; Stone & Carson, 2017). Recognizing the insidious, implicit character of this bias could help laypeople and scholars counter its effects.

## ACKNOWLEDGMENTS

We thank Curie Cha and Ankush Maheshwari for their contribution to this research.

## FUNDING INFORMATION

This research was not supported by any external funding source.

## AUTHOR CONTRIBUTIONS

I. B.: Conceptualization; Formal analysis; Methodology; Writing – original draft. A. S.: Data curation; Investigation; Software; Writing – review & editing.

## DATA AVAILABILITY STATEMENT

All data are provided in a supplementary data file: https://doi.org/10.1162/opmi.a.7.

## Supplementary Material




