# Supplementary material for: Are You a Closet Dualist? Evidence From Brief Implicit Association Task
Source: Open Mind (Camb). 2025 Jul 26;9:864–84. doi: 10.1162/opmi.a.7 (PMC12373452; doi:10.1162/opmi.a.7)
Supplement: Supplementary file 2 [file opmi-09-864-s002.docx]

**Dualism is an implicit cognitive bias:**

**Evidence from Brief Implicit Association task**

***Supplementary materials***

Iris Berent^1^***

Alexzander Sansiveri^1^

^1^Northeastern University

*Corresponding author: Iris Berent

Address for correspondence:

Iris Berent, Ph.D

Department of Psychology

Northeastern University

125 Huntington Ave.

Boston, MA 02115

[I.berent@northeastern.edu](mailto:I.berent@northeastern.edu)

**Detailed Methods**

**Experiment 1**

**Design.** Each experimental block of trials featured all 16 experimental targets along with a category-attribute pair (e.g., BODY-OBJECT). Thus, within a block, half of the targets belonged to the featured category or attribute pair—these are the ***focal*** targets. The other half were ***non-focal*** targets, as they did not belong to either the category or attribute.

Block order was counterbalanced across four experimental lists using a Latin-square design. Thus, each participant was assigned to a single list, featuring a total of 128 experimental trials (4 blocks x 2 repetitions x 16 targets). Table S1 provides the full design.

As noted (in the Main Text), Experiment 1 featured the categories BODY (hand, leg, fingers, toes) and MIND (knowledge, belief, ideas, thoughts), and the attributes OBJECT (chair, table, ball, pen) and STUFF (water, sand, snow, mud).

**Table S1**. The design of the experimental lists.

**Practice trials.** To master the task, participants were given multiple sets of practice.

*Practice block.* At the beginning of the experimental session, participants were given a practice block, consisting of two sections. The first section was designed to familiarize participants with the attributes only. To this end, participants were given four trials, featuring two of the OBJECT attributes (chair, table) and two of the STUFF attributes (water, sand).

Next, participants were given a second practice section, in which they classified targets with respect to *both* category and attribute. The attribute here was OBJECT and the practice category was MAMMAL. Targets representing the (focal) OBJECT and (non-focal) STUFF attributes were as in the experimental session (chair, table, ball, pen; water, sand, snow, mud). Four other targets represented the MAMMAL category (dog, cat, human, cow) and four conveyed the (non-focal) category BIRD (robin, bluebird, pigeon, chicken).

In both practice sections, participants were given feedback on their response accuracy (“Correct”, “Incorrect”). Likewise, slow responses (longer than 4 seconds) triggered a warning message (“No response detected”). Once participants’ accuracy reached 75%, they were administered the experimental session, consisting of eight experimental blocks.

*Experimental practice.* Each experimental block commenced with four practice trials, featuring the focal attribute of the subsequent experimental trials (hereafter, the *Experimental practice)*. For example, if the experimental block featured OBJECT as the focal attribute (and STUFF as non-focal), the practice trials would feature those same focal and non-focal attributes; feedback (for accuracy and speed) was as in the previous practice block. Next, participants were given the instructions for the experimental trials (presented on the computer screen); the experimental trials followed. Altogether, then, each experimental block featured 20 trials (4 practice trials and 16 experimental trials).

**Procedure**  is described in the Main Text. Figure S1 illustrates the display in experimental trials.

**Figure S1.** An illustration of a trial display in Experiments 1-.


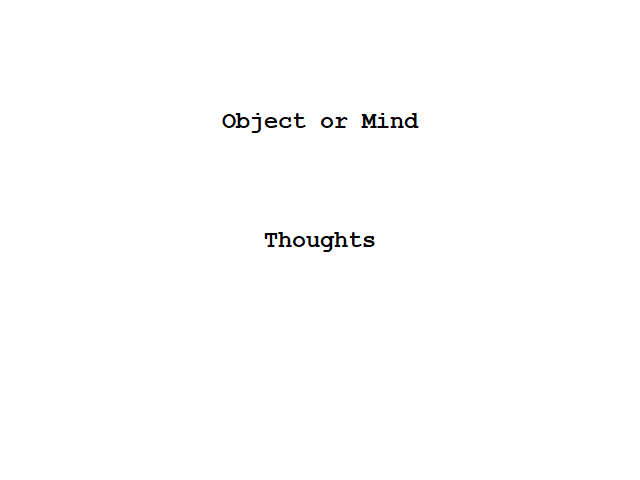


**Experiment 2**

The structure of this experiment (experimental trials and practice) was as Experiment 1, except that the MIND category now featured four emotions: fear, surprise, anger, happiness (instead of the epistemic states, featured in Experiment 1). The BODY targets were as in Experiment 1, except that plural nouns (toes, fingers) were replaced by singulars (toe, finger). The attributes OBJECT and STUFF remained unchanged, as in Experiment 1.

**Experiment 3**

The practice block was as in Experiments 1-2, except that the (non-focal) category BIRD was replaced with FISH (cod, trout, tuna, and salmon).

Each experimental block now featured two experimental practice sections (rather than one, previously), for a total of 8 trials. The design of this practice and feedback were exactly as in Experiments 1-2.

As noted, Experiment 3 also included a survey, administered to 20 Prolific workers. The instructions and questionnaires are provided in Appendix I.

**Experiment 4**

Participants in this experiment first took the BIAT task (as in Experiment 3); they were next administered two explicit measures of Dualism: the mind-body task and the afterlife task. Appendix I provides the full materials and instructions.

The experiment also included a third explicit measure, inviting participants to express their beliefs about the mind-body divide by choosing one of seven diagrams, expressing the distance between bodies and minds (Forstmann & Burgmer, 2015). As expected, the mean response was below the scale’s midpoint (M=2.43, SE=0.13). Upon further analysis, however, we determined that this measure cannot be clearly interpreted as evidence for either “physicalism” or “dualism”, as the middle of the scale still separated bodies and minds (as two tangential circles). For this reason, we excluded the data from this measure.

**Experiment 5**

As noted, in Experiment 5, the trial structure was changed, to contrast the category/attribute combination (e.g., SOLID, BODY) with the word“neither”, Figure S2 illustrates the display.


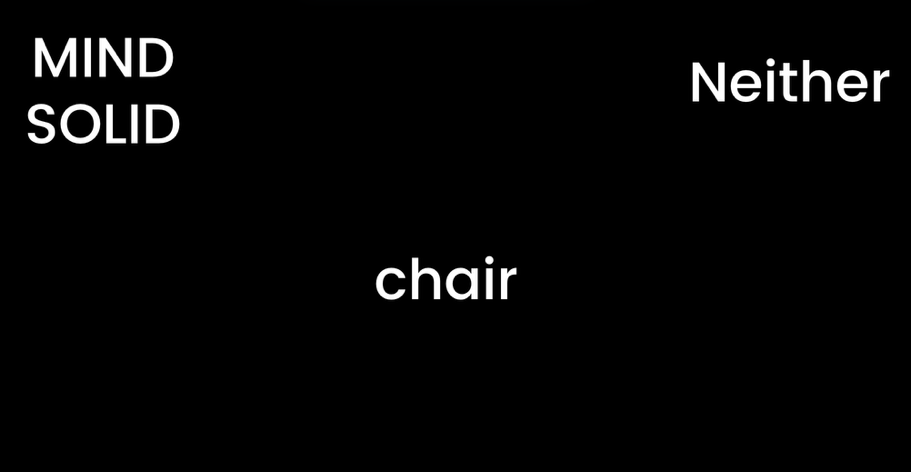


**Figure S2.** An illustration of a trial display in Experiments 5-6.

Figure S3 offers a glimpse of the links between the link between explicit and implicit Dualism at the individual subject level. Implicit Dualism is capture as follows. In d’: (body_solid-body_airy)+(mind_airy-mind_solid); in RT, we do the converse (body_airy-body_solid)+(mind_solid-mind_airy). Below,we plot between individual participans’ scores of implicit Dualism and their explicit Dulaism scores, captured by their mean rating in the mind-body and afterlife tasks.


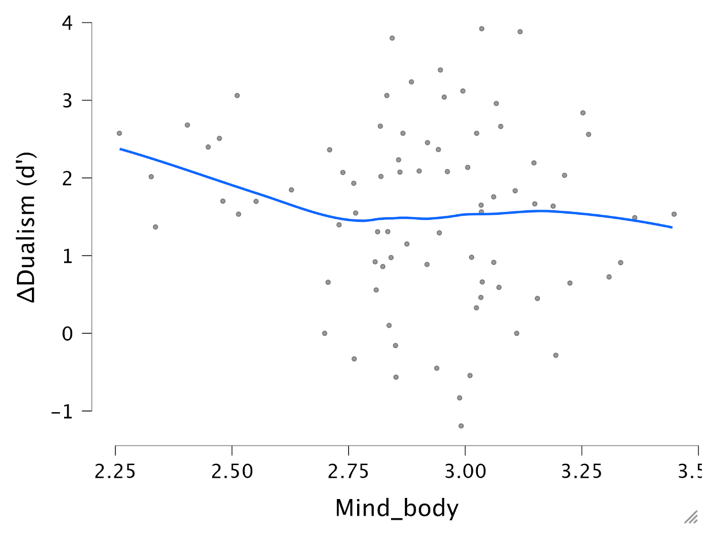

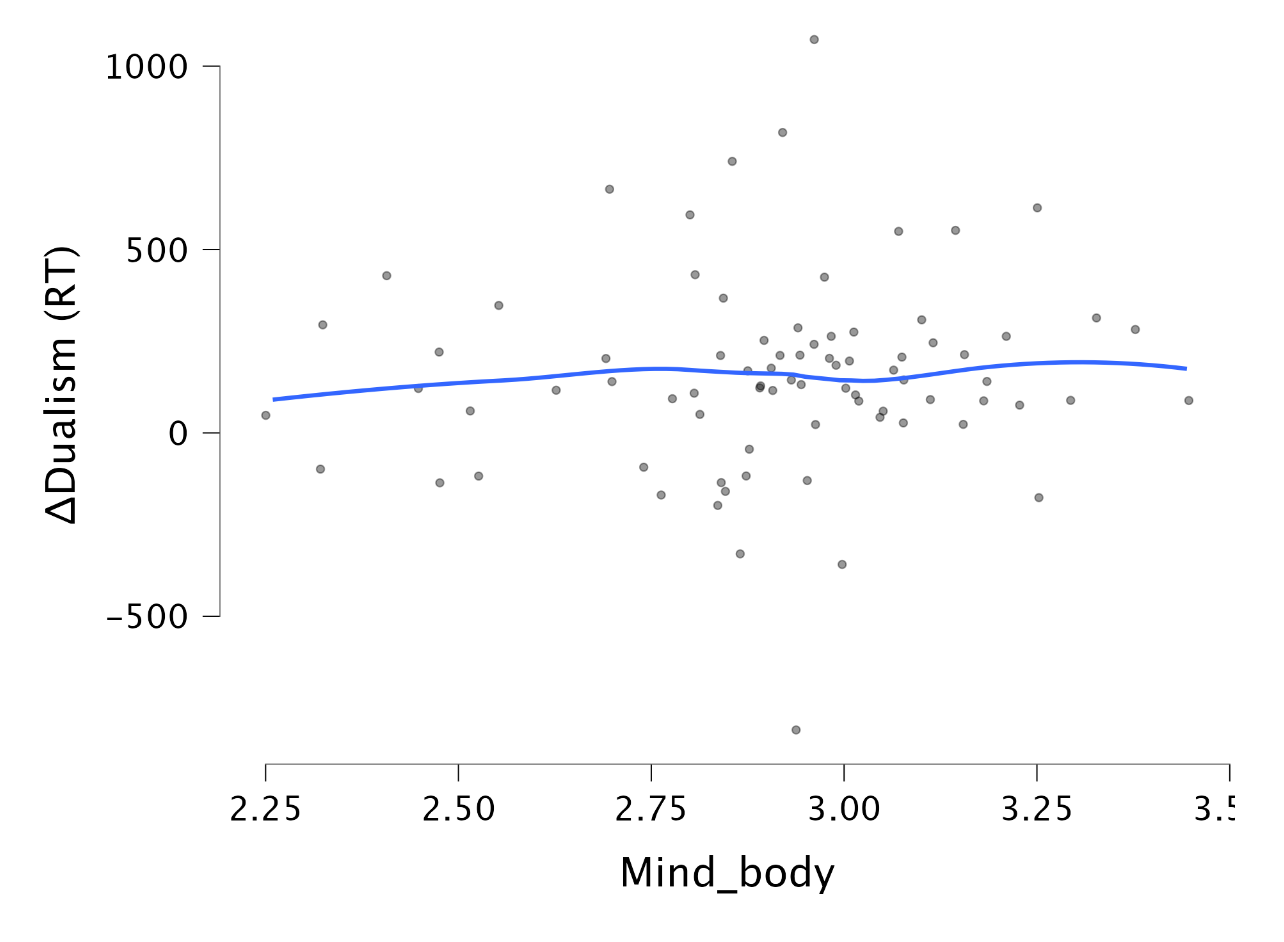

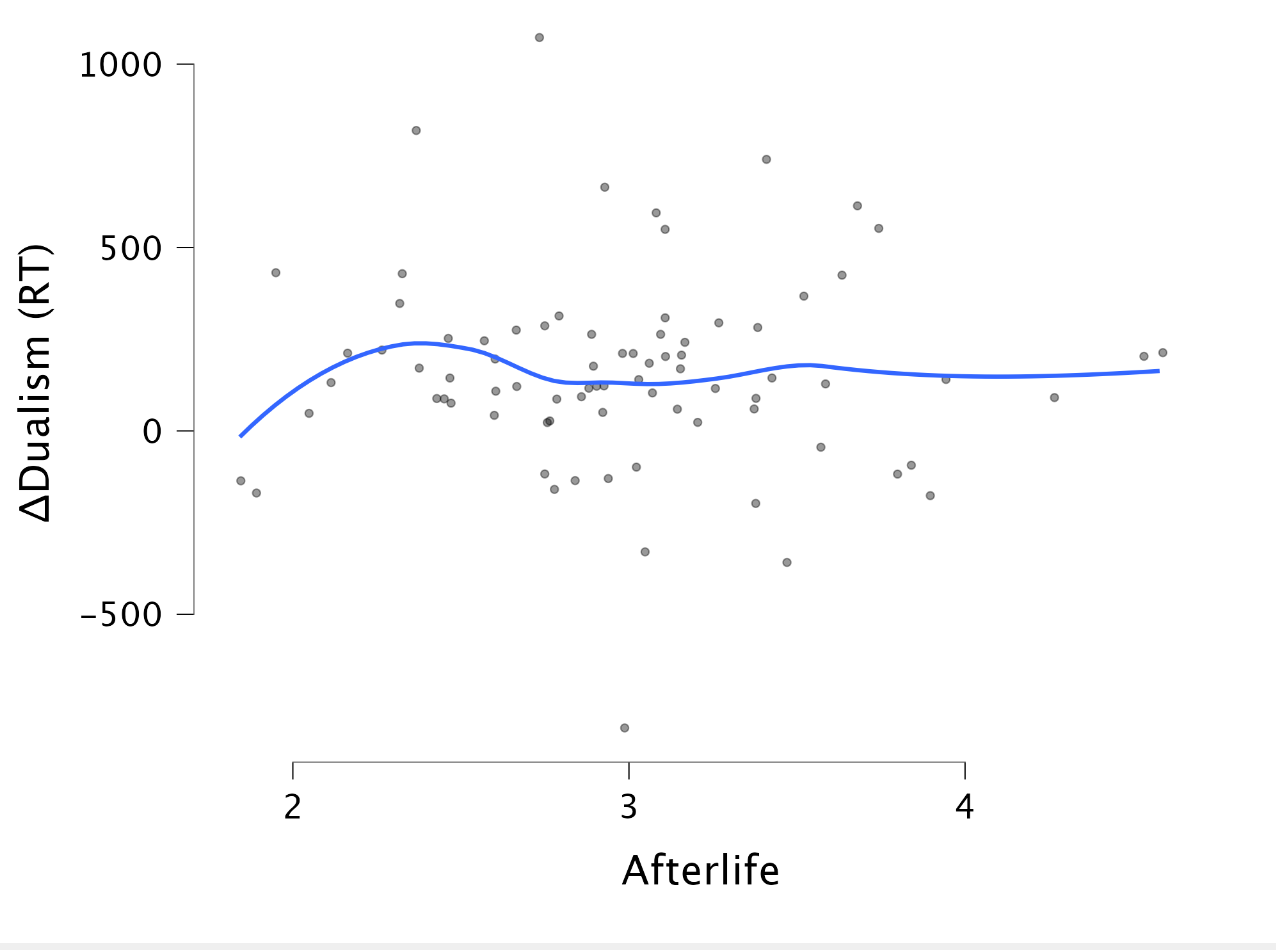

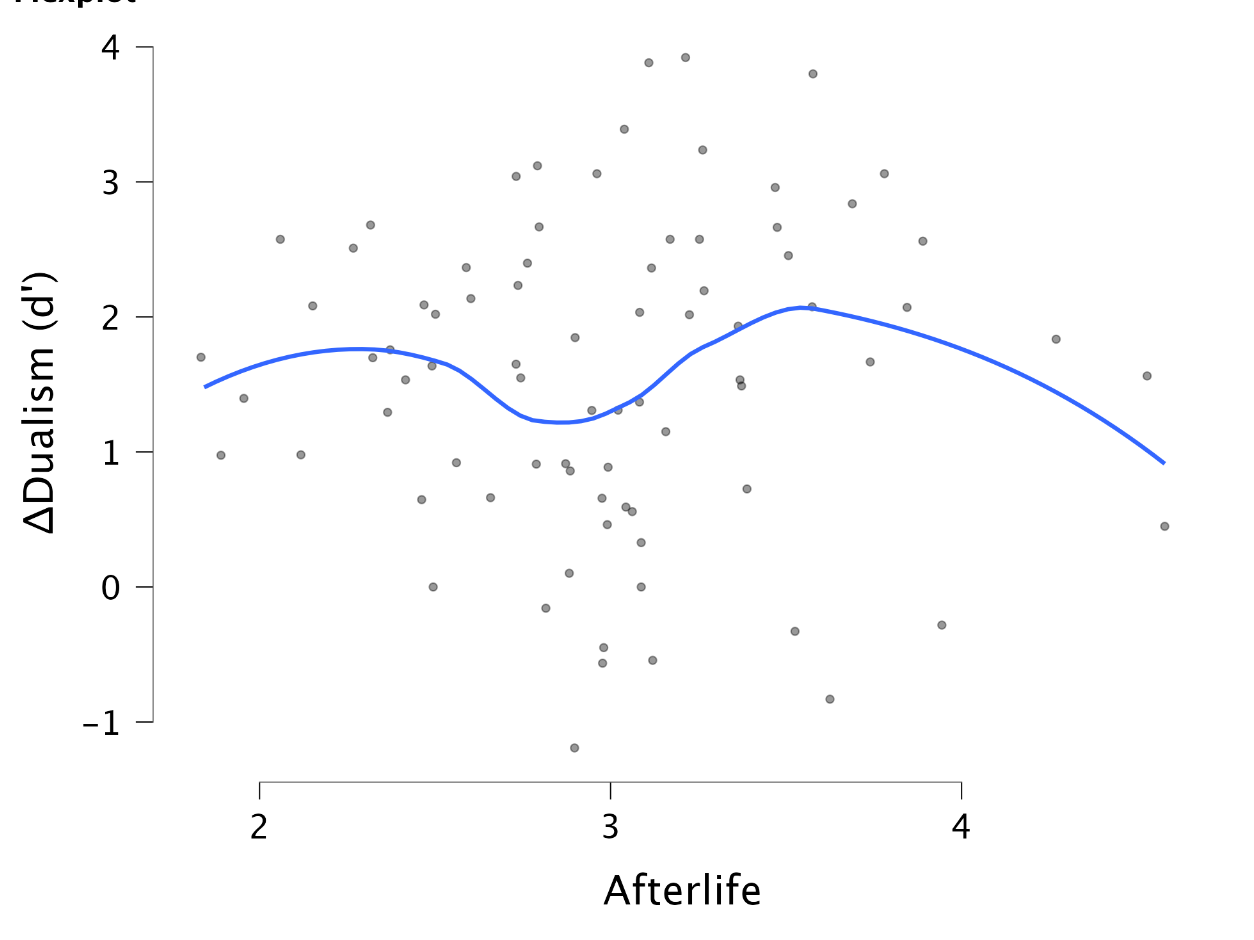


**Figure S3.** The correlations between explicit and implicit Dualism.

**Experiment 6**

Experiment 6 (not reported in the main text) presents another pre-registered replication of Experiments 4-5. The design is identical to Experiment 5, except that the mind-body question was omitted due to an experimenter error. The remainder of the results fully replicate those reported in Experiment 5. 144 Northeastern students took part in this experiment.

**a. Explicit Dualism**. When Dualism was gauged explicitly, in terms of afterlife beliefs, participants showed no evidence for explicit Duaism. In fact, their mean afterlife beliefs was significantly lower than the “neutral” midpoint (M=2.94, t(143)=3.49, p<.001).

**b. Implicit Dualism.**  When Dualism was assessed implicitly (using BIAT), however, the results offered strong evidence for Dualism. The 2 Dualism x 2 Attribute interaction was significant in both dprime (F(1, 143)=212.35, p<.001, η²p=0.598) and RT (F(1, 143)=64.00, p<.001, η²p=0.309).


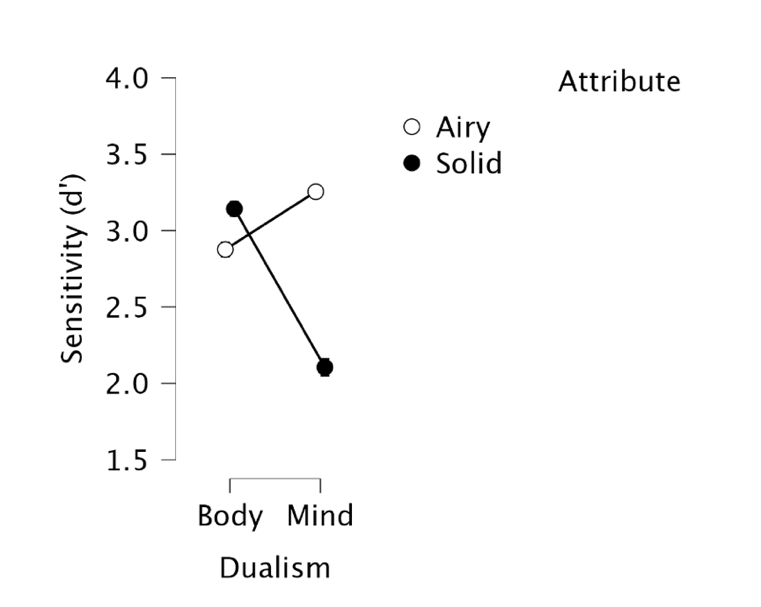

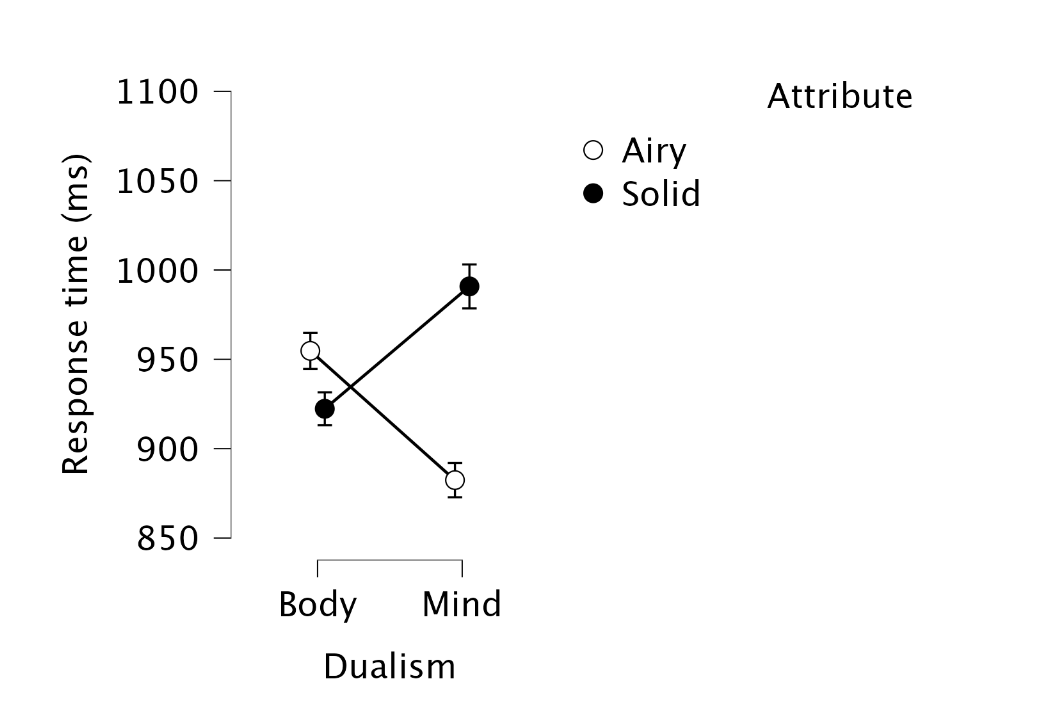


**Figure S4.** Mind-Body responses in Experiment 6.

The interaction (Figure S4) emerged because responses to BODY were more sensitive (F(1,143)=16.39, p<.001) and faster F(1,143)=4.94, p=.03) with SOLID (compared to AIRY), whereas response to MIND were more sensitive F(1,143)=290.83, p<.001) and faster F(1,143)=39.49, p<.001) with AIRY (compared to SOLID)

**c. Non-Dualist participants**. We next turned to analyze the responses of participants who explicitly rejected the afterlife (i.e., mean <3); there was a total of 85 such participants. Because, in this experiment, we did not evaluate these participants attitudes about the mind-body question, we can only describe them as non-Dualist (as opposed to physicalist, specifically). Overall, their results matched those of the entire sample.

As might be expected, these participants strongly denied that the afterlife exists (M=2.81, t(84)=13.09, p<.001). But when given the BIAT, they showed implicit evidence for Dualism (Figure S5). The 2 Dualism x 2 Attribute interaction was significant in sensitivity(F(1, 84)=108.123, p<.001, η²p=0.563) and response time (F(1, 84)=31.51, p<.001, η²p=0.273).

The interaction emerged because the response to BODY was more sensitive(F(1,84)=5.92, p=.03) with SOLID relative to AIRY; in response time, this contrast was not significant (F(1,84)=2.78, p=.10). In contrast, response to MIND were more sensitive (F(1,84)=172.776, p<.001)and faster (F(1,84)=20.07, p<.001)with AIRY relative to BODY. These results establish that participants can show implicit Dualism even when they reject the persistence of the mind after death.


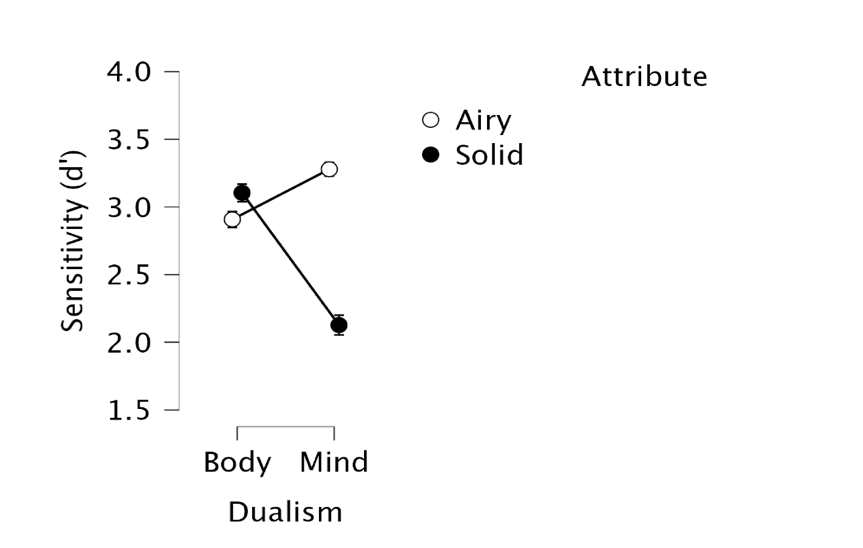

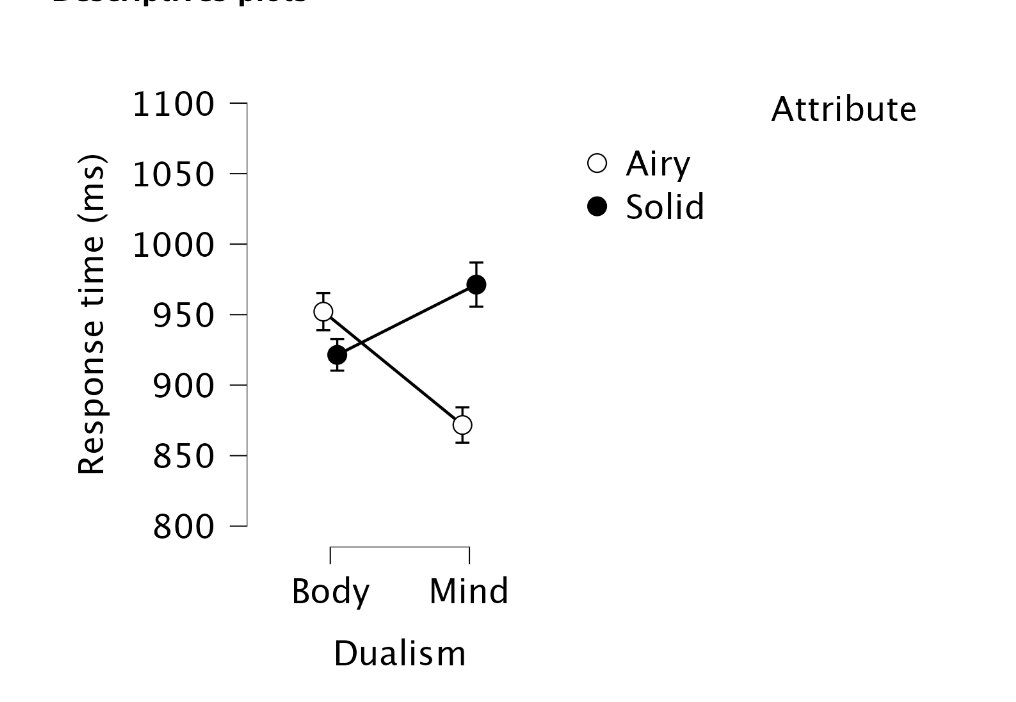


**Figure S5.** Mind-Body responses for non-Dualist participants in Experiment 6.

**Appendix I**

Instructions

In this study, we ask you to rate words on a five-point scale. For each word, please consider whether it is material or ethereal.

***Material** things have the physical properties of matter.
***Ethereal**things **aren't** material or physical.

Please try to use the entire five-point scale. 

Thank you!


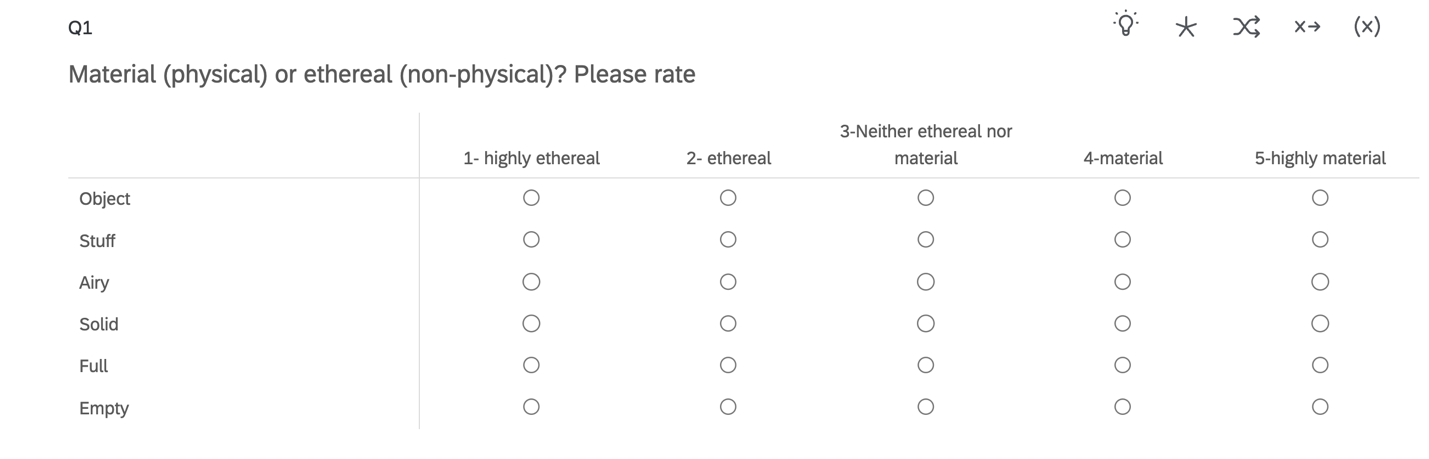


**Appendix II**

**Instructions and materials in the explicit measures of Dualism**

Please evaluate each statement on a 1-5 scale; if you strongly agree press 5; if you strongly disagree, press 1.


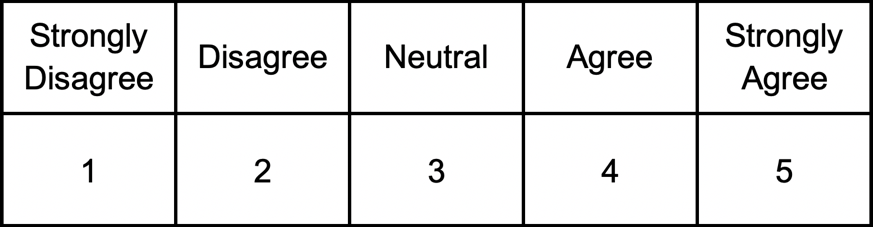


1. **Body-mind**

| **ID** | **Questions** | **Reverse**  **coding** |
| --- | --- | --- |
| 1 | The mind is not part of the brain but it affects the brain. | no |
| 2 | When I imagine a scene in my mind, I am in a state that will forever be beyond explanation by science. | no |
| 3 | When I use the word "mind," it is just a shorthand term for the complicated things that my brain does. | yes |
| 4 | The mind is a special form of energy (currently unknown to man) that is in contact with the brain and affects it. | no |
| 5 | Minds are in principle independent of bodies, to which they are only temporarily "attached." | no |
| 6 | Hundreds of years in the future when we know how brain states and thoughts are related, it might be possible for a physiologist to measure my brain states and know what I am thinking. | yes |
| 7 | Sometimes when I give reasons for my behavior, those reasons are wrong. That is, my behavior can be affected by things that I am not aware of. When this happens I might give a "reason" for my behavior that is wrong. | yes |
| 8 | Perhaps it will never make sense to talk about computers having emotions, but some time in the future it may be the case that computers will think as well as humans. | yes |
| 9 | Mental processes cause changes in brain processes. | no |
| 10 | When people talk about their minds they are really just talking about what their brains seem to be doing. Talk about the "mental is really just a shorthand for brain processes that we are not aware of. | yes |
| 11 | The fact that I can know my own thought processes (that I can introspect) means that my thought processes cannot be just brain processes. | yes |
| 12 | Just as we no longer talk of witches, in the future when we know in detail how brains work, we may not talk about minds anymore. | yes |
| 13 | My mind is the thing that causes me to behave as I do. | no |
| 14 | Knowledge of the mind will forever be beyond the understanding of sciences like physics, neurophysiology, and psychology. | no |
| 15 | For each thought that I have, there exists a certain state that my brain is in. | yes |
| 16 | The mind is a nonmaterial substance that interacts with the brain to determine behavior. | no |
| 17 | Hundreds of years in the future, when we know much more about the brain and behavior, we might change the way we talk about our behavior and our minds. That is, we might find better ways to talk about our thoughts, feelings, and emotions. | yes |
| 18 | The "self" that I introspect about controls both the mind and the brain. | no |
| 19 | Minds are inside brains but are not the same as brains. | no |
| 20 | When we say that a person has a "creative mind" this just means that the person tends to produce things that people judge as creative. The statement really has nothing to do with the person's mind. | yes |
| 21 | Some mental processes have no connection to brain processes. | no |
| 22 | In a hundred years or more, it might make sense to refer to a computer as having a mind. | yes |
| 23 | My consciousness will survive the disintegration of my physical body. | no |
| 24 | Not much would be lost if we dropped the word "mind" from our vocabularies. For example, rather than say "I made up my mind" a person might say "My brain decided." Although this might sound funny at first, no meaning would be lost. | yes |
| 25 | Mental processes are the result of activity in the nervous system. | yes |
| 26 | The mind and the brain are two totally separate things. | no |
| 27 | We talk of the sun rising but we all know that the sun does not rise but instead the earth turns. This is a case of our language not responding to changes in physical knowledge. Some neurophysiologists think this might also be the case for our language about the mental. For instance, if we had adequate physiological knowledge it might be possible to say "My C-fibers are firing!" instead of "I'm in pain!" Just as phrases like "The sun is rising" are expendable (not needed, since the sun really doesn't rise) some mental terms may be eliminated or drastically changed in the future when we have better physiological knowledge. | yes |

1. **Afterlife**

Please evaluate each statement on a 1-5 scale; if you strongly agree press 5; if you strongly disagree, press 1.


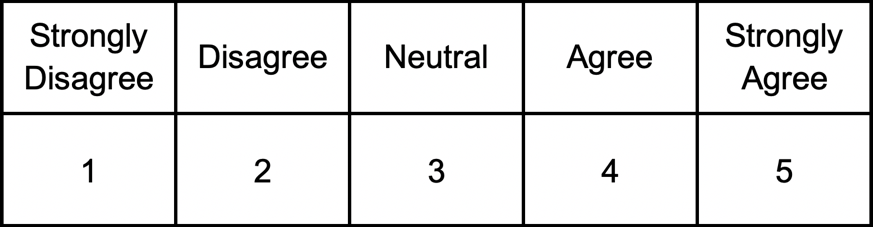


| **ID** | **Questions** | **Reverse coding** |
| --- | --- | --- |
| 1 | Earthly existence is the only existence we have. | yes |
| 2 | In the premature death of someone close, some comfort can be found in knowing that in some way the deceased is still existing. | no |
| 3 | Humans die in the sense of "ceasing to exist." | yes |
| 4 | There must be an afterlife of some sort. | no |
| 5 | We will never be united with those deceased whom we knew and loved. | yes |
| 6 | Some existentialists claim that when people die they cease to exist: I agree. | yes |
| 7 | The following statement is true: "There is no such thing as life after death." | yes |
| 8 | Millions of people believe in a life after death: they are correct in so believing. | no |
| 9 | Enjoy yourself on earth, for death signals the end of all existence. | yes |
| 10 | The life we lead now is but a pebble cast upon the sands of our future lives. | no |
| 11 | Religiously associated or not, belief in an afterlife will never be shown to be true, for afterlives are nonexistent. | yes |
| 12 | The following statement is true: "Humans must enter into some sort of existence after death, for the end of our earthly lives cannot wipe us from existence into nothingness." | no |
| 13 | When a human dies, that something called "life" dies with him or her. | yes |
| 14 | The idea of there existing somewhere some sort of afterlife is beyond my comprehension. | yes |
| 15 | The millions killed by Hitler passed from existence into nothingness. | no |
| 16 | Many scientists believe in a life after death: they are right, there is one. | no |
| 17 | There is supportive evidence for the existence of an afterlife. | no |
| 18 | Death ends all forms of life forever. | yes |
| 19 | The existence of an afterlife can never be scenically demonstrated, for it is impossible to prove a "figment of someone's imagination." | yes |

Forstmann, M., & Burgmer, P. (2015). Adults Are Intuitive Mind-Body Dualists. *Journal of Experimental Psychology: General, 144*(1), 222-235.
